# Clavicle refractures after hardware removal: are there risk factors? A retrospective cohort study

**DOI:** 10.1007/s00068-025-02794-x

**Published:** 2025-02-21

**Authors:** Franziska Kessler, Yannik Kalbas, Jan Hambrecht, Victoria Wlach, Sascha Halvachizadeh, Roman Pfeifer, Hans-Christoph Pape, Felix Karl-Ludwig Klingebiel, Christian Hierholzer

**Affiliations:** 1 Harald-Tscherne Laboratory for Orthopaedic and Trauma Research, University Hospital Zurich, University of Zurich, Ramistr. 100, 8091 Zurich, Switzerland; 2 Department of Trauma Surgery, University Hospital Zurich, University of Zurich, Ramistr. 100, 8091 Zurich, Switzerland; 3 Department of Trauma, Hand, and Reconstructive Surgery, University Hospital Frankfurt, Goethe University, Frankfurt/Main, Germany; 4 Department of Plastic Surgery and Hand Surgery, Cantonal Hospital Aarau, Aarau, Switzerland

**Keywords:** Clavicle fracture, Refracture, Working length

## Abstract

**Purpose:**

Removal of symptomatic hardware after fracture fixation is common, especially in patients with clavicle fracture. Yet, refracture after hardware removal is a relatively common complication in those patients. The aim of this study was to identify risk factors for clavicle refractures that could be influenced by the surgical treatment provided.

**Methods:**

All patients from a level one trauma center from 2017 to 2022 were screened for eligibility. Inclusion criteria included hardware removal after plate osteosynthesis of the clavicle, age ≥ 18 years, and signed informed consent. Groups were stratified according to occurrence of refracture: no-refracture (NR) vs. refracture (R). Nearest-neighbor matching in a ratio of 5:1 was performed. Parameter investigated included baseline demographics, fracture characteristics and surgical treatment details. A subgroup analysis of only clavicle shaft fractures was performed.

**Results:**

Sixty patients were included with 50 patients in Group NR and 10 in Group R. Baseline characteristics were comparable between the groups. A subgroup analysis on shaft fractures revealed that a significantly larger working length (number of empty screw holes adjacent to the fracture site) in Group NR (1.94 ± 0.85) compared to the refracture group (1.20 ± 0.92) (p = 0.042). Logistic regression yielded an inverse correlation with the number of empty screw holes to the fracture site and the occurrence of refractures (OR 0.369, 95% CI 0.132–0.873; p = 0.035). Time in situ, lag screw application, plate positioning and the total amount of screws did not affect either of the groups.

**Conclusion:**

An increased working length in patients with clavicle shaft fractures might be a protective measure for occurrence of refractures after hardware removal.

## Introduction

The current surgical gold standard for the treatment of displaced midshaft clavicle fractures is open reduction and internal fixation (ORIF) using plate osteosynthesis [1]. Lateral clavicle fracture can be treated either with ORIF using a hook plate, an anatomic locking plate, or using minimally invasive acromioclavicular joint reconstruction (MINAR) technique [2, 3]. The advantage of surgical fixation is early functional rehabilitation [4]. Plate osteosynthesis in clavicle fractures often leads to symptomatic hardware due to the anatomical conditions of the clavicle (little amount of underlying soft tissue, stress point for backpacks and bags), so that implant removal is often requested by patients over the course of treatment [4]. In a recent study, our team evaluated the indications and outcomes of hardware removal of the upper extremity in which refractures of the clavicle occurred in 3.7% after hardware removal. The most common indication was symptoms of unspecific discomfort (53.7%), followed by pain (29.3%) and limited range of motion (ROM) (7.3%), as well as a combination of both (pain and ROM: 9.8%) [5]. The study demonstrated that patients experiencing unspecific discomfort exhibited an absence of benefit from hardware removal, in contrast to those with pain or restricted range of motion, who demonstrated a positive outcome after removal. It is imperative to acknowledge the surgical procedure for removal and the variable clinical course that ensues. In accordance with these factors, the hardware should be maintained in situ unless requested by the patient [6].

It is reported in the literature that precontoured plates and an anteroinferior plate positioning might result in a lower removal rate [7, 8]. However, after removal of the clavicle osteosynthesis material, refractures can occur. In the literature, occurrence of clavicle refracture is reported between 2–7% and results in an unfortunate clinical course for the patient [5, 9, 10]. One hypothesis that aims to explain why the clavicle is more susceptible to refractures is the thin cortical structure in combination with the residual screw holes that remain following implant removal [10]. Furthermore, the clavicle experiences a variety of forces, including compression, bending and torsion during in routine activities [9]. In the case of fractures of the upper extremity, it is generally recommended that hardware should be removed after a minimum of 18 months in order to reduce the risk of subsequent refractures [11]. Also, the literature has addressed the topic of an initially reduced load on the bone following implant removal [12].

Therefore, we aimed to investigate risk factors for clavicle refractures to identify patients that might not benefit from or qualify for hardware removal.

We hypothesized that early implant removal and fracture location at the transition zone from lateral to mid-shaft may constitute risk factors for clavicle refracture.

## Methods

### Setting

The study was conducted in accordance with the Declaration of Helsinki [11] and the Swiss Cantonal Ethics Committee (BASEC No. 2023-0129). The reporting of this retrospective cohort study was performed in according to the STROBE guidelines (STROBE: Strengthening the Reporting of Observational Studies in Epidemiology) [13]. The study was performed in a Swiss level 1 trauma center (Department of Traumatology, University Hospital Zurich).

### Study population

The patients included in our study underwent hardware removal after ORIF of the clavicle between January 2017 and December 2022. Patient identification was conducted via an automated search of the clinical information system. Only patients with signed informed consent were included in the initial search. Inclusion criteria were: hardware removal of plate osteosynthesis after ORIF of the clavicle in the defined time-period, age ≥ 18 years, signed informed consent. Exclusion criteria were: peri- implant fracture of the clavicle, fixation methods other than plating (e.g. titanium elastic nail (TEN)), pathological fracture and incomplete data set. Groups were stratified according to occurrence of refracture.

### Definitions and classification

Patient demographics and comorbidities were extracted from the patient files at the time of accident. Fractures were classified according to the AO/OTA classification system. Radiological images were screened by two authors independently to identify fracture locations (shaft and lateral location). Fractures located at the middle-lateral thirds transition were additionally marked for further analysis, which we from now on refer to as “fractures in the transition zone” [14]. The total amount of empty screw holes bridging the fracture were counted as it displays the working length and is indicative of the rigidity of the construct. Time in situ was defined as the number of days between plate osteosynthesis and hardware removal.

### Indication for hardware removal

In all patients who underwent hardware removal, radiographical proof of complete fracture consolidation had been obtained prior to the surgery. Indications for hardware removal included symptomatic hardware with either unspecific discomfort or pain and impaired range of motion of the adjacent shoulder joint. Timing of implant removal was determined based on a comprehensive assessment of radiological and clinical signs of fracture consolidation, assessed by an experienced surgeon, and the interval since the initial treatment.

Following the application of hook plates in ORIF procedures, the removal of implants was initiated after a minimum of three months, for midshaft fractures treated using plating osteosynthesis the implant removal was planned after a minimum of 18 month.

### Statistics

Continuous data are presented with mean and standard deviation, categorical variables with numbers and percentages. Statistical analysis was performed in R using the “Stat” and “Tableone” packages [15]. Matching was performed in R using the “MatchIt” package and figures were computed using the “ggplot2” package. MS-Excel was used for data-visualization. Data was visually tested for normality using histograms. Binary data were assessed using a two-sided Fisher’s exact test, non-binary categorical data using Chi-squared test and continuous, normally distributed parameters with the Student’s t-test. Significance level was set at 0.05. Patients from both groups (no refracture vs. refracture) were matched using a nearest-neighbor approach in a 5:1 ratio for age and gender via propensity score matching. A subgroup analysis of the patients with shaft fracture was performed.

### Data extraction

Data were organized and stored using Microsoft Excel on password-protected in-house computers. The rate of missing data was extremely low, as the parameters of interest were defined a priori, with particular emphasis on those routinely entered our clinical system. In case of missing data, the parameter was marked as N/A (not available) for that patient and excluded from the analysis.

## Results

**Fig. 1:**
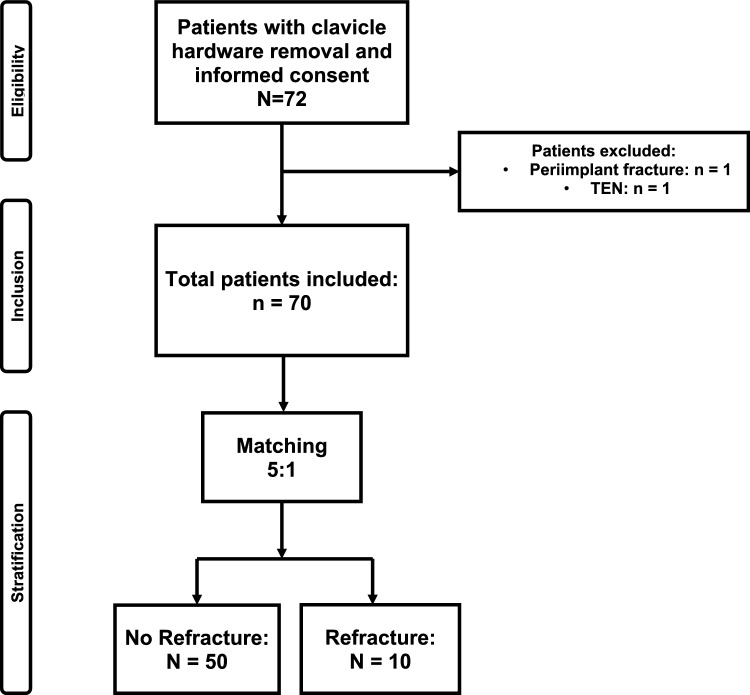
Flowchart of patient inlcusion

From initially 72 patients, sixty patients were included in the study after the matching process. 50 patients from Group NR were matched with the 10 patients in Group R (Fig. 1).

The mean age for both groups was 40 years (NR: 39.66 ± 13.55 years vs. R: 40.60 ± 13.41 years, p = 0.843) with a mean BMI of 24 (BMI; NR: 24.31 ± 2.93 vs. R: 24.03 ± 4.40). Four out of 10 patients in group R were female (40%) compared to 16% group NR (p = 0.101). The American Society of Anesthesiologists risk classification (ASA) mean score in group NR was 1.78 ± 0.77 and 1.50 ± 0.53 in group R (p = 0.185). Fourteen patients (29.2%) in group NR were active smokers compared to five patients (50%) in group R (p = 0.270). None of the patients in either group was diabetic. Only one patient (2%) in group NR had a diagnosed vascular disease (p = 1). These baseline demographics with additional standard mean differences (SMD) are displayed in Table 1.

**Table 1:** Patient characteristics

	No-refracture (N = 50)	Refracture (N = 10)	p-value	SMD
Demographics				
Age, mean (± SD)	39.66 (± 13.55)	40.60 (± 13.41)	0.843	0.07
Sex (female), n (%)	8 (16.0)	4 (40.0)	0.101	0.554
BMI, mean (± SD)	24.31 (± 2.93)	24.03 (± 4.40)	0.862	0.073
Comorbidities				
ASA, mean (± SD)	1.78 (± 0.77)	1.50 (± 0.53)	0.185	0.417
Smoking (yes), n (%)	14 (29.2)	5 ( 50.0)	0.270	0.436
Diabetes (yes), n (%)	0 (0)	0 (0)	N/A	0
Vascular disease (yes), n (%)	1 (2.0)	0 (0)	1	0.204

Refractures only occurred in the shaft area (n = 10, 100%), which makes up for 64% (n = 32) in group NR (p = 0.025). Hardware removal in patients with lateral shaft fractures was not related to any refractures and makes up for 36% in group NR (Table 2).

**Table 2:** Fracture and treatment characteristics

	No-refracture	Refracture	p-value
Fracture characteristics			
Fracture location, n (%)			0.025
Shaft	32 (64.0)	10 (100.0)	
Lateral	18 (36.0)	0 (0)	
Classification, n (%)			0.154
15.2A	10 (20.8)	3 (30.0)	
15.2B	4 (8.3)	3 (30.0)	
15.2B und 15.3C	1 (2.1)	0 (0.0)	
15.2C	16 (33.3)	4 (40.0)	
15.3A	14 (29.2)	0 (0.0)	
15.3C	3 (6.2)	0 (0.0)	
Fracture in transition zone, n (%)	8 (17)	0 (0.0)	0.327
Treatment characteristics			
Plate positioning, n (%)			0.312
Anterior	25 (50.0)	7 (70.0)	
Superior	25 (50.0)	3 (30.0)	
Lag screw application, n (%)	10 (20.0)	4 (40.0)	0.222
Total amount screws, mean (± SD)	6.86 (± 1.55)	7.60 (± 1.17)	0.105
Empty holes to fracture site (working length), mean (± SD)	1.39 (± 1.08)	1.20 (± 0.92)	0.577
Time in situ, mean (SD) (days)	551.42 (± 274.64)	712.60 (± 210.75)	0.054

**Fig. 2:**
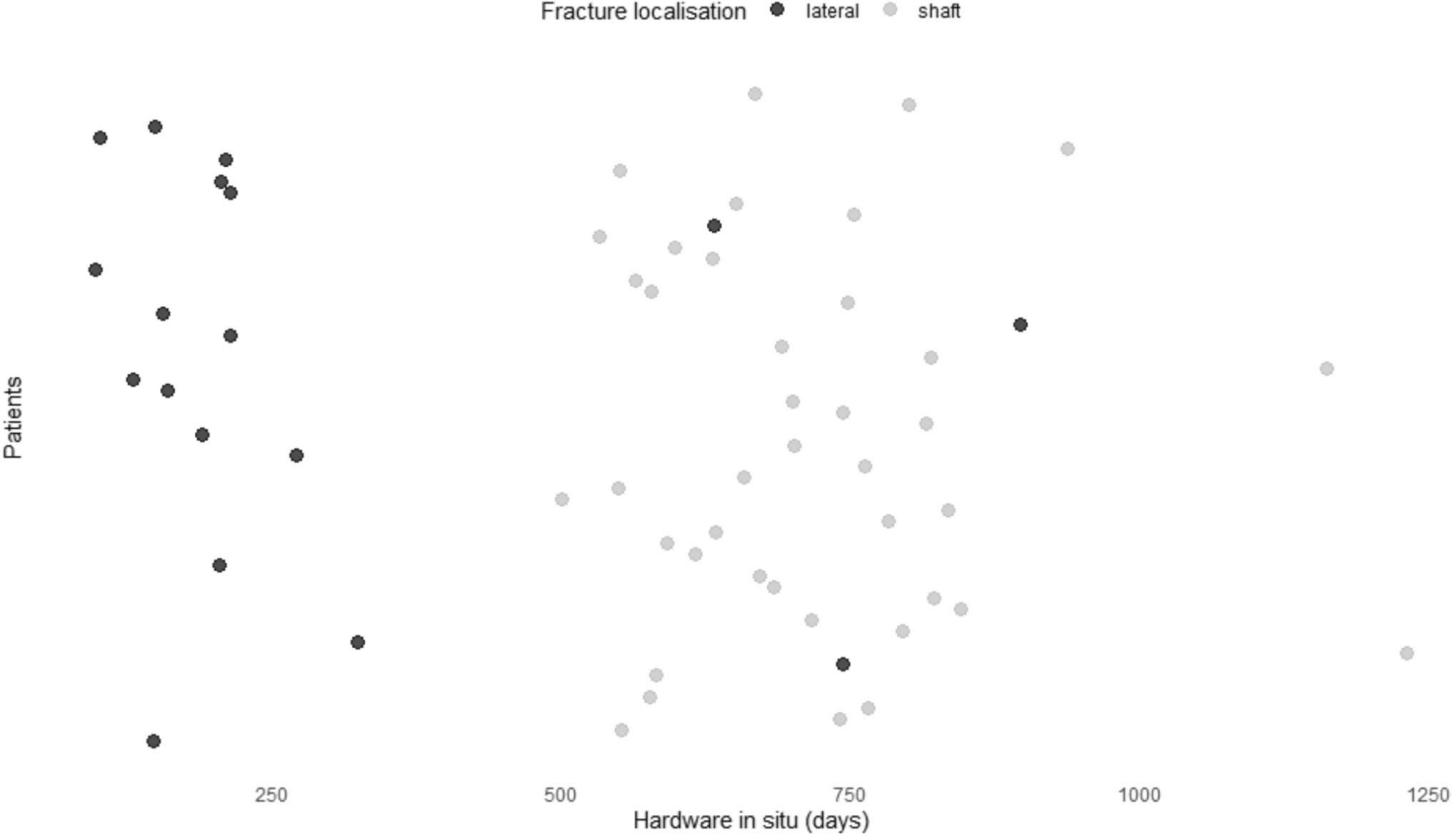
Visualization of time in situ according to the fracture side

Eight patients (17%) with initial fracture in the transition zone were among the patients without refracture whereas none in the refracture presented this fracture characteristic (p = 0.154). In group NR half of the patients received superior plate positioning and the other half received anterior plate positioning compared to 70% (n = 7) with anterior and 30% (n = 3) with superior positioning in the refracture group (p = 0.312). Twenty percent (n = 10) of patients in group NR received lag screw application compared to 40% (n = 4) in the refracture group (p = 0.222). The mean number of screws used in the in group NR was 6.86 ± 1.55 compared to 7.60 ± 1.17 in group R (p = 0.105). A mean of 1.39 ± 1.08 empty holes bridging the fracture (working length) was present in the NR group compared to 1.20 ± 0.92 in the refracture group (p = 0.577). The mean time in situ in the NR group was 551.42 ± 274.64 days compared to 712.60 ± 210.75 days (p = 0.054). Yet, the patients with lateral fracture location received a consequent earlier hardware removal based on the treatment protocol (Fig. 2).

Based on these results, a subgroup analysis of only the shaft fractures was conducted. The baseline demographics of this specific cohort are displayed in Table 3.

**Table 3:** Subgroup analysis (only clavicle shaft fractures)

Shaft fractures only	No-refracture (N = 32)	Refracture (N = 10)	p-value
Demographics			
Age, mean (± SD)	37.84 (10.91)	40.60 (13.41)	0.564
Sex (female), n (%)	|4 (12.5)	4 (40.0)	0.075
BMI, mean (± SD)	24.60 (2.61)	24.03 (4.40)	0.721
Comorbidities			
ASA, mean (± SD)	1.81 (0.79)	1.50 (0.53)	0.175
Smoking (yes), n (%)	10 (33.3)	5 (50.0)	0.457
Diabetes (yes), n (%)	0 (0.0)	0 (0.0)	N/A
Vascular disease (yes), n (%)	1 (3.2)	0 (0.0)	1
Fracture characteristics			
Classification, n (%)			0.546
15.2A	10 (33.3)	3 (30.0)	
15.2B	4 (13.3)	3 (30.0)	
15.2C	16 (53.3)	4 (40.0)	
Fracture in transition zone, n (%)	7 (24.1)	0 (0.0)	0.158
Treatment characteristics			
Plate positioning, n (%)			1
Anterior	24 (75.0)	7 (70.0)	
Superior	8 (25.0)	3 (30.0)	
Lag screw application, n (%)	9 (28.1)	4 (40.0)	0.700
Total amount screws, mean (± SD)	7.28 (1.30)	7.60 (1.17)	0.476
Empty holes to fracture site (working length), mean (± SD)	1.94 (0.85)	1.20 (0.92)	0.042
Time in situ, mean (SD) (days)	714.80 (132.52)	712.60 (210.75)	0.976

**Fig. 3:**
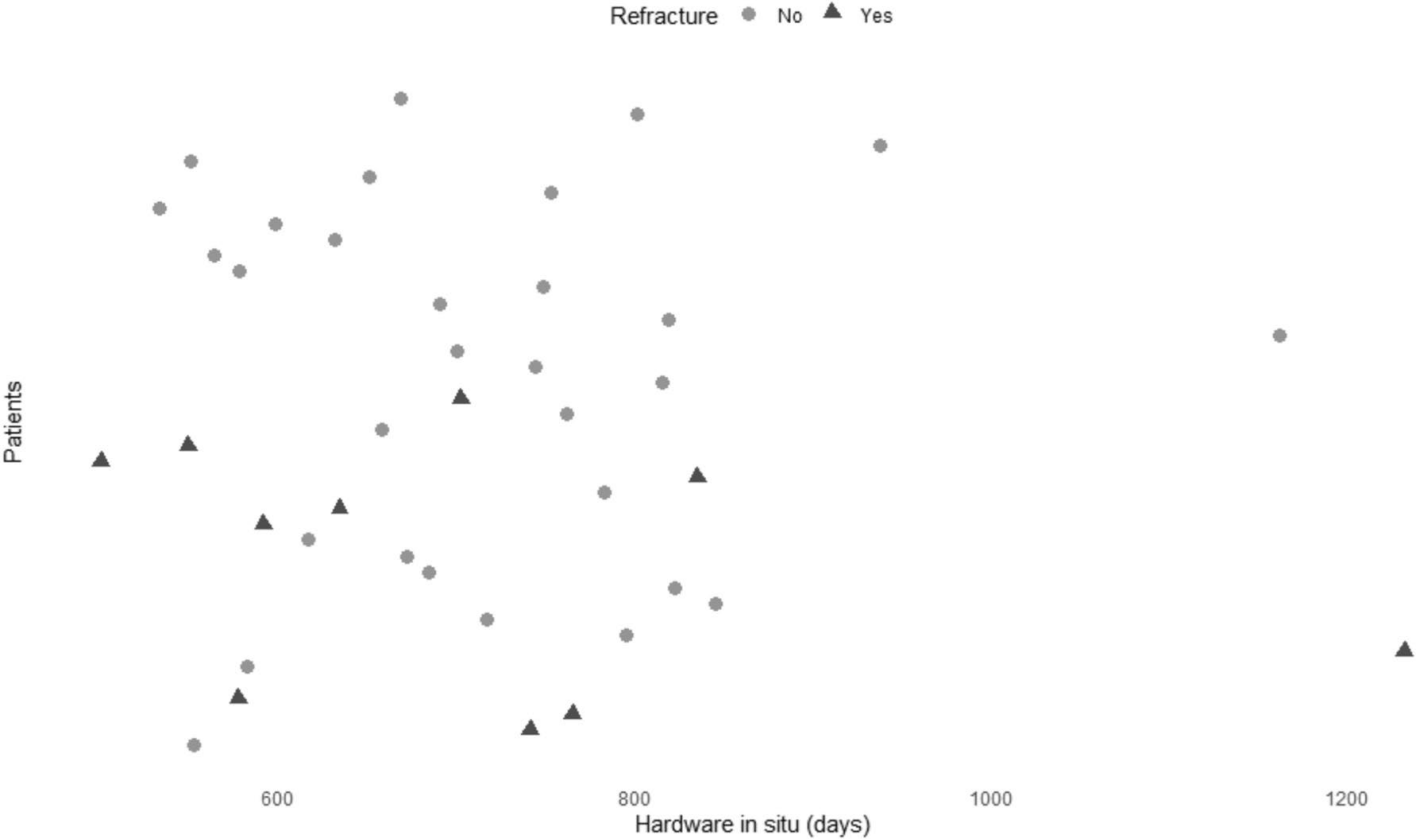
Timepoint of refractures after hardware removal after clavicle shaft fracture

Only midshaft fractures, only fractures of type 15.2A-C were analyzed and no significant differences between the group characteristics was observed (p = 0.546). Fracture location in the transition zone was identified in group NR in 24.1% (n = 7) whereas in the refracture group no fracture localization in the transition zone was found. Plate positioning was also comparable with 75/70% (NR/R) anterior and 25/30% (NR/R) superior plating (p = 1). 28.1% (n = 9) of the patients in group NR received additional lag screw application compared to 40% (n = 4) in group R. There was no significant difference in the total number of screws with 7.28 ± 1.30 screws in group NR and 7.60 ± 1.17 screw in group R (p = 0.476). Significantly more empty holes (greater working length) were present in the NR group with 1.94 ± 0.85 empty holes bridging the fracture in group NR and 1.20 ± 0.92 in group R (p = 0.042). The mean time in situ was similar in the groups with 714 ± 132.52 days in the NR and 712.60 ± 210.75 in the R group (0.976) (Fig. 3).

Logistic regression revealed that the number of empty holes to the fracture site as a protective factor for refractures which represents a longer working length (OR 0.369, 95% CI 0.132–0.873; p = 0.035). The remaining characteristics presented in Table 3 were also investigated, but these were found to be non-significant in the linear model. This data analysis suggests, that an additional empty hole may decrease the risk of a refracture by approximately 63%.

## Discussion

In our study we evaluated multiple demographic, clinical and radiological parameters and their impact on the risk for refracture following removal of osteosynthetic plate. Based on the results of our study we conclude the following main findings:

- Shaft fractures of the clavicle are more prone to refracture after hardware removal compared to lateral fractures.
- The time in situ in our study cohort had no impact on refractures.
- More empty holes adjacent to the fracture site in clavicle shaft fractures representing greater working length may be a protective factor concerning refractures. This constitutes a greater working length.

A recent study by Tsai et al. investigated risk factors for refracture of midshaft clavicle fractures in an eastern population and identified female sex and lower BMI as risk factors [10]. We could not reproduce these findings in our cohort as in our study the BMI in our main and subanalysis was comparable between the groups and located more to the upper limit of a normal BMI (BMI = 24). However, the authors did not investigate radiographical risk factors. In our study we could not detect refractures in patients with lateral clavicle fractures. Despite the relatively small sample size of this group, it is still remarkable as the hook plates that were predominately used for this fracture pattern which are already removed after a few months. Good et al. report similar results in their study, which investigated the outcome after hook plate osteosynthesis, In their cohort only 2 out of 36 patients (5.6%) suffered a refracture after hardware removal which was caused by adequate trauma [2].

Regarding the influence of the time in situ on refractures is ambivalent in the literature. The same holds true for the role of the anatomic location. Tsai et al. did not identify the time in situ as a risk factor in their study [10]. However, they reported that implant removal was performed at earlier time points after around one year following the initial osteosynthesis. In our study cohort, the mean time in situ was approximately two years. As our study investigated a different time horizon compared to Tsai et al., our results in this regard may therefore just be comparable to a certain extent. In patients with forearm fractures, a hardware removal after less than 18 months is reported to be more prone to refractures [11]. In contrast, in patients with proximal femur fractures, patients who received implant removal after 2–3.5 years presented a higher general complication rate with refractures being the most common among them [16]. The “perfect” time point for hardware removal is therefore still under investigation but timeframes such as “too early” and “too late” seem to exist in this context. The period of up to 18 weeks after implant removal seems to be especially vulnerable reported as the bone might require time to heal in the area of the former screws [17]. Consequently, an initial reduction in stress on the respective anatomical area may prove beneficial. Yet, this is an aspect that we did not evaluate in our study.

It is discussed in the literature that regarding the role of the working length, stiffness of the osteosynthetic construct may result in unstructured cortical bone formation with less rigidity and reduced callus formation even after the process of bony consolidation is terminated [18, 19]. In constructs with an increased working length, micromotion of the fracture zone may stimulate more bone deposit and callus formation. This hypothesis is supported by our data as we identified a higher number of empty screw holes adjacent to the fracture site to be a protective factor for refractures. The effect of increased stiffness leading to bone atrophy has already been described as early as 1976 [20]. Additionally, Stoffel et al. reported that sufficient working length should be respected as it improves fracture healing and callus formation in fractures [21]. Therefore, it is tempting to hypothesize that a less rigid construct may be beneficial for the healing process in clavicle fractures. This might lead to an improved bone quality that might be more resistant to suffering a refracture after hardware removal [21].

### Strengths and limitations

Our study is limited by the relatively small sample size from a single center and we might therefore be underpowered to detect additional parameters of interest. In our institution, research may only be conducted on patients with a personally signed informed consent for using de-identified data for research practice which is not routinely signed by the patients. Yet, we aimed for a normal distribution and comparable baseline characteristics between the groups using the nearest-neighbor matching approach.

## Conclusion

We conclude that time in situ in our cohort did not affect the incidence of refractures significantly. However, an increased working length in the initial plate osteosynthesis may improve the bone healing process and bone stability. Therefore, it may be advisable not to place screws in the holes closest to the fracture site to allow micromotion at the fracture site and to promote secondary bone healing. Prospective or randomized studies with a larger sample size as well as biomechanical studies are required to gain more insight.

## Data Availability

Data, (further) materials and code can be requested individually from the author team. The team of authors reserves the right to evaluate and decide individually to hand out the requested data/materials/code.

## Abbreviations

ASA: American Society of Anesthiosologists
BMI: Body Mass Index
MINAR: Minimally-invasive AC-joint reconstruction
ORIF: Open reduction internal fixation
ROM: Range of motion
SMD: Standard mean difference
TEN: Titanium elastic nail
